# Assessing the Burden of Illness Associated with Acquired Generalized Hypoactive Sexual Desire Disorder

**DOI:** 10.1089/jwh.2021.0255

**Published:** 2022-05-16

**Authors:** James A. Simon, Amod Athavale, Rahul Ravindranath, Nandini Hadker, Amama Sadiq, Michelle Lim-Watson, Laura Williams, Julie Krop

**Affiliations:** ^1^ Department of Obstetrics and Gynecology, George Washington University and IntimMedicine™ Specialists, Washington, District of Columbia, USA.; ^2^ TRINITY, Waltham, Massachusetts, USA.; ^3^ AMAG Pharmaceuticals, Inc., Waltham, Massachusetts, USA.

**Keywords:** female sexual dysfunction, hypoactive sexual desire disorder, mental health, quality of life, premenopausal

## Abstract

**Background:**

Hypoactive sexual desire disorder (HSDD), which affects ∼10% of women in the United States, is defined as the persistent or recurrent deficiency/absence of sexual desire accompanied by personal distress. Although HSDD impacts patient quality of life and interpersonal relationships, the disorder often goes unaddressed or untreated. Recent studies of the burden of illness in women with HSDD, especially premenopausal women, are limited.

**Materials and Methods:**

A 45-minute web-based survey was designed to investigate the experience of women seeking treatment for HSDD and the impact of this disorder on several psychosocial aspects of women's lives. Women were recruited from an online panel of patients who participated in research studies for compensation. Validated questionnaires assessed sexual function (Female Sexual Function Index) and health-related quality of life (12-Item Short Form Survey [SF-12]), including mental and physical component scores.

**Results:**

A total of 530 women, aged ≥18 years, diagnosed with acquired generalized HSDD were included in the study. Premenopausal women indicated greater overall HSDD symptom burden compared with postmenopausal women. Patients with HSDD reported lower SF-12 scores compared with the general population. A multivariable regression analysis demonstrated that psychosocial factors influencing the burden of HSDD, including interference with their relationship with their partner (β = −0.18; *p* < 0.005), mental and emotional well-being (β = −0.23; *p* < 0.005), and household and personal activities (β = −0.23; *p* = 0.02), negatively affected SF-12 mental component scores.

**Conclusions:**

HSDD symptom burden was found to be negatively and statistically significantly associated with patients' mental health; the impact was greater among premenopausal women compared with postmenopausal women.

## Introduction

Female sexual dysfunction (FSD) encompasses physiological, psychological, and social components, including several sexual concerns that can be distressing to women. In the United States, the most prevalent form of FSD is hypoactive sexual desire disorder (HSDD).^1,2^ The *Diagnostic and Statistical Manual of Mental Disorders, Fourth Edition, Text Revision* (DSM-IV-TR) defines HSDD as a disorder characterized by an absence or deficiency of sexual desire or fantasies accompanied by marked distress that is not due to a coexisting medical or psychiatric condition, problems with the relationship, or the effect of a medication.^3^ In 2013, HSDD and female sexual arousal disorder (FSAD) were absorbed into a single condition termed female sexual interest and arousal disorder (FSIAD) in the *Diagnostic and Statistical Manual of Mental Disorders, Fifth Edition* (DSM-5).^4^

Consistent with the DSM-IV-TR definition of HSDD, the International Society for the Study of Women's Sexual Health consensus panel characterized HSDD as the persistent or recurrent deficiency or absence of sexual desire and/or receptiveness to erotic stimulation, accompanied by distress.^2^ A cross-sectional population-based study of the prevalence of FSD (PRESIDE) showed that ∼1 in 10 U.S. women suffer from low sexual desire and associated distress: HSDD affects 8.9% of women aged 18–44 years, 12.3% of women aged 45–65 years, and 7.4% of women aged 65 years and older.^5^ Although HSDD impacts women's health-related quality of life (HRQoL), the disorder often goes unaddressed or untreated.^6^

Women may delay or not seek treatment for HSDD for various reasons, including the belief that their sexual difficulties are temporary and will resolve or their experience is a normal part of aging or long-term relationships, an aversion to discussing what they see as a private or embarrassing matter, and uncertainty about where to seek help.^7^ Additionally, physicians who frequently treat HSDD, such as primary care providers (PCPs) and obstetricians/gynecologists (OB/GYNs), are often hesitant to initiate discussions about sexual health due to concerns of causing discomfort in their relationships with their patients as well as the physicians' own underlying attitudes regarding sexuality.^1^

PCPs and OB/GYNs may also be less confident in their ability to treat HSDD due to limited awareness of screening tools and treatment options, as well as a lack of training in human sexuality during their formal medical education.^1,8^

The impact of HSDD on HRQoL was previously evaluated only in postmenopausal women.^9^ To gain further insight into the burden of HSDD, this study investigated the experience of women seeking treatment for HSDD and characterized the impact of this disorder on several psychosocial aspects of women's lives. It also assessed the unmet medical needs relevant to this condition in these women.

## Materials and Methods

### Study design

We designed a 45-minute, web-based cross-sectional survey, approved by an institutional review board, that explored the patient experience, including the physical, social, mental, economic, and emotional burden related to HSDD. Participants were anonymously recruited from the Curizon online patient research panel, an entity that individuals join for the purpose of participating in research studies for compensation. When they enter the panel, participants are asked about all diagnosed conditions they have and are then sorted into the database.

Pretest evaluations were conducted via cognitive interviews among participants to minimize potential response error and to evaluate the quality of responses. One pretest was conducted for each age group of interest: 18–30, 31–45, 46–60, and 61–80 years. Screening criteria for the pretest evaluation were identical to participant eligibility criteria for this study. Participants who qualified for the pretest evaluation were asked to identify questions or response options that were unclear, not comprehensive, or potentially erroneous. No sources of response error were identified.

Participants were offered reward points equivalent to ∼$28 in exchange for their time. Those who met the eligibility criteria and completed the study survey were included in the study analyses. The survey was administered from August 24, 2018, through September 18, 2018.

### Survey description

The final survey, approved by the University of Mississippi's Institutional Review Board, consisted of customized questions that assessed demographics, HSDD symptom details, diagnosis and initial perceptions, management of HSDD, and disorder impact on HRQoL. Validated questionnaires assessed sexual function (Female Sexual Function Index [FSFI] scale^10^) and HRQoL (12-Item Short Form Survey [SF-12]).^11^ The contextual relevance of the survey was validated and strengthened with input from discussions with sexual medicine experts.

### Participant eligibility

The study recruited women aged ≥18 years who were in a stable monogamous relationship for ≥6 months. Menopausal status was classified according to Stages of Reproductive Aging Workshop criteria^12^; HSDD diagnosis was based on self-reported physician diagnosis or symptoms of HSDD in accordance with the Decreased Sexual Desire Screener (DSDS) (Supplementary Table S1).^13^ Exclusion criteria included prior hysterectomy or bilateral oophorectomy.

### Statistical analyses

All statistical analyses were performed using IBM SPSS Statistics 24 (IBM, Armonk, NY, USA).

#### Descriptive analyses

Means were calculated for continuous variables; frequencies and percentages were calculated for categorical variables. Student's *t*-tests with Bonferroni corrections and chi-squared tests were conducted as appropriate. The Bonferroni correction was applied to account for multiple comparisons and was performed by multiplying the uncorrected *p*-value with the number of comparisons. If this multiplied *p*-value is <0.05, the comparison was deemed to be statistically significant.

#### Multivariable linear regression analyses

Two independent multivariable linear regression analyses were performed. A multivariable linear regression model was used to evaluate the relationship between psychosocial aspects of burden and the overall burden of HSDD; independent variables were psychosocial factors, and the dependent variable was the overall burden of HSDD. Mean scores for the independent variables were calculated and included in multivariable linear regression analyses. The internal consistency of the mean scores for “mental and emotional well-being” (Cronbach's α = 0.909) and “household and personal activities” (Cronbach's α = 0.968) was validated.

Another multivariable linear regression model assessed the relationship between psychosocial factors (independent variables) and the SF-12 mental component score (MCS) (dependent variable). Both multivariable regression models included the following control variables: age, race, education, marital status, and social desirability bias (as measured by egoistic and moralistic response tendencies).^14^

## Results

### Sample description and demographics

Of the 32,178 participants in the panel who were initially recruited for the web-based survey, 1,508 (4.6%) received HSDD diagnoses, 1,108 (3.4%) had a physician diagnosis of HSDD, and 418 (1.3%) qualified based on the DSDS criteria. Among these 1,508 participants, 857 (50%) met the eligibility requirements to participate in the study, and 530 (62%) completed the survey and were included in the study analyses. The majority of participants were premenopausal, married, Caucasian, college graduates, and in a heterosexual relationship (Table 1).

**Table 1.** Participant Demographics

Demographic	Participants (*n* = 530),* n *(%)
Age, years	
18–45	307 (58)
46–60	180 (34)
61–80	43 (80)
Menopause status	
Premenopausal	409 (77)
Postmenopausal	121 (23)
Comorbid conditions	
Depression	201 (38)
Anxiety	180 (34)
Race/ethnicity	
Caucasian	398 (75)
African American	64 (12)
Hispanic/Latina	37 (7)
Asian	21 (4)
Other	10 (2)
Marital status	
Married	376 (71)
Living with partner	69 (13)
Single (never married)	53 (10)
Divorced	16 (3)
Other	16 (3)
Highest level of education	
College graduate or above	212 (40)
Some college or AA degree	191 (36)
High school	117 (22)
Middle school	10 (2)
Current employment status	
Full time	266 (50)
Homemaker	108 (20)
Part-time	74 (14)
Retired	32 (6)
Disabled	30 (6)
Unemployed/seeking employment	15 (3)
Full/part-time student	5 (1)
Reason for current employment status	
Not at all due to my decreased sexual desire	419 (79)
In small part due to my decreased sexual desire	58 (11)
In large part due to my decreased sexual desire	32 (6)
Entirely due to my decreased sexual desire	21 (4)
Nature of stable relationship	
Heterosexual	503 (95)
Homosexual	27 (5)
Spoken to a doctor/therapist?	
Yes	398 (75)
No	132 (25)
Who started the conversation?	
I initiated the conversation	435 (82)
My doctor/physician initiated	95 (18)

AA, Associate of Arts.

### Unmet medical needs in diagnosis and management of HSDD

Among all participants, 75% indicated that they had spoken to a doctor or therapist about their HSDD symptoms, whereas the remaining 25% had not (Table 1). Of those who had spoken to a doctor or therapist, 82% of participants initiated the conversation with their health care provider (HCP) (Table 1). On average, it took 10 months for participants to approach a physician after experiencing initial symptoms of HSDD, and those participants experienced ∼2 months' delay in diagnosis with HSDD (Supplementary Fig. S1).

The most frequently cited reasons for not approaching a doctor/therapist were assumptions that HSDD symptoms were normal for their age (35%) and embarrassment (30%) (Supplementary Fig. S2). Diagnosis of HSDD was often determined by the same doctor/therapist respondents initially approached (*n* = 324) (Supplementary Table S2). Interestingly, 21% of women who initially approached a PCP were ultimately diagnosed by a gynecologist. Symptoms were initially dismissed or misdiagnosed by doctors/therapists as depression or anxiety in up to 44% of cases (Supplementary Fig. S1). Of note, 18% of the participants who experienced delay in diagnosis indicated that their HCPs did not know HSDD was a medical condition, whereas 13% of those participants were uncomfortable discussing HSDD as a medical condition.

Regarding management and treatment of HSDD, participants reported that HCPs were far more likely to recommended nonpharmacological treatments (Supplementary Fig. S3). The commonly recommended nonpharmacological treatments were lubricants/moisturizers (52%), relaxation/meditation/yoga (43%), psychotherapy (39%), weight loss/diet/exercise (32%), and herbal or homeopathic products (19%). At the time of this study, flibanserin was the only treatment approved by the U.S. Food and Drug Administration (FDA) for premenopausal women with HSDD,^15^ and it was prescribed to 7% of survey participants.

### Sexual health assessment

**FIG. 1.**
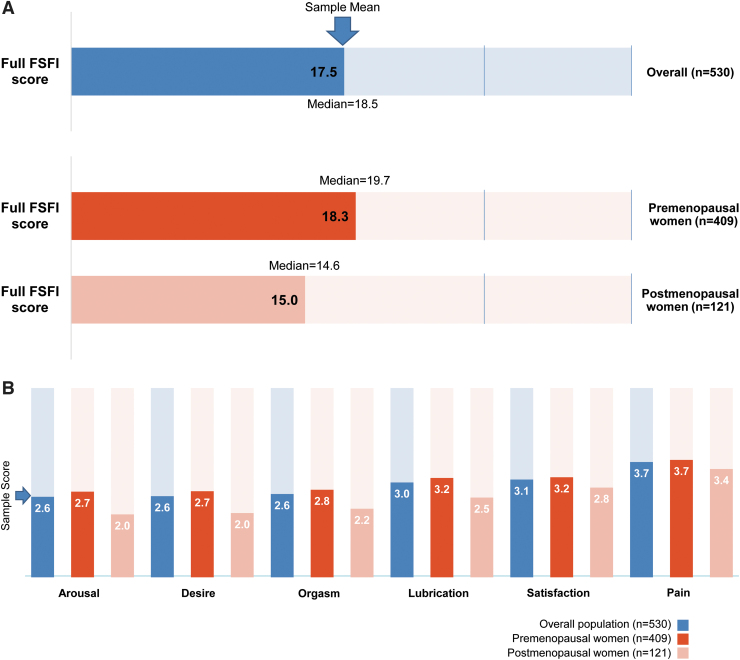
Impact of HSDD on sexual function (*n* = 530). Comparisons between premenopausal and postmenopausal women were analyzed using Student's *t*-tests. **(A)** Mean and median FSFI total score among all respondents and by menopausal status. Maximum score (perfect sexual functioning) = 36. Scores ≤26.55 indicate female sexual dysfunction. **(B)** Mean FSFI domain scores among all respondents and by menopausal status. Maximum score (perfect sexual functioning) = 6. For each of the six domains (arousal, desire, orgasm, lubrication, satisfaction, and pain), differences between premenopausal and postmenopausal were statistically significant (all *p* < 0.001). The difference in overall FSFI score between the two groups was also statistically significant (*p* < 0.001). FSFI, Female Sexual Function Index; HSDD, hypoactive sexual desire disorder.

A total score of 36 on the FSFI scale^10^ indicates no sexual dysfunction, whereas scores ≤26.55 indicate sexual dysfunction.^16^ The mean FSFI total score of all participants was 17.5 ± 7.1, indicating that sexual functioning was significantly below normal (Fig. 1A). Furthermore, the mean FSFI total score in postmenopausal women (15.0) was significantly lower (*p* < 0.001) than that in premenopausal women (18.3).

When assessed for each aspect of sexual function, participants reported a score of 2.6 in the arousal, desire, and orgasm domains out of a maximum score of 6 for each domain of the FSFI (Fig. 1B). Postmenopausal women exhibited significantly lower scores on the arousal (*p* < 0.001), desire (*p* < 0.001), orgasm (*p* < 0.001), and lubrication (*p* < 0.001) domains compared with premenopausal women. Sexual activity was measured by the mean number of events of caressing, foreplay, and vaginal intercourse/penetration; participants reported a 50% decrease in frequency of all three events after the onset of HSDD symptoms (data not shown). However, there were no statistically significant differences in the frequency of those sexual activities between premenopausal and postmenopausal women.

### Impact of HSDD on social relationships

To understand the burden of HSDD on women's social relationships, the impact of HSDD on relationships with partners/spouses, friends, family, and co-workers was assessed. Participants were also asked to report the impact of HSDD on their overall lives. Responses were scored on a scale of 0–100, with 0 indicating no interference in the independent variables listed above and 100 indicating extreme interference. Notably, among divorced women (3% of total participants), 27% reported that their divorce was primarily due to decreased sexual desire.

Premenopausal women with HSDD showed significantly greater interference across all social relationships compared with postmenopausal women (*p* = 0.01 for all relationships). Correspondingly, participants aged 61–80 years with partners (*n* = 42) experienced the least interference across all social relationships compared with other age groups.

**FIG. 2.**
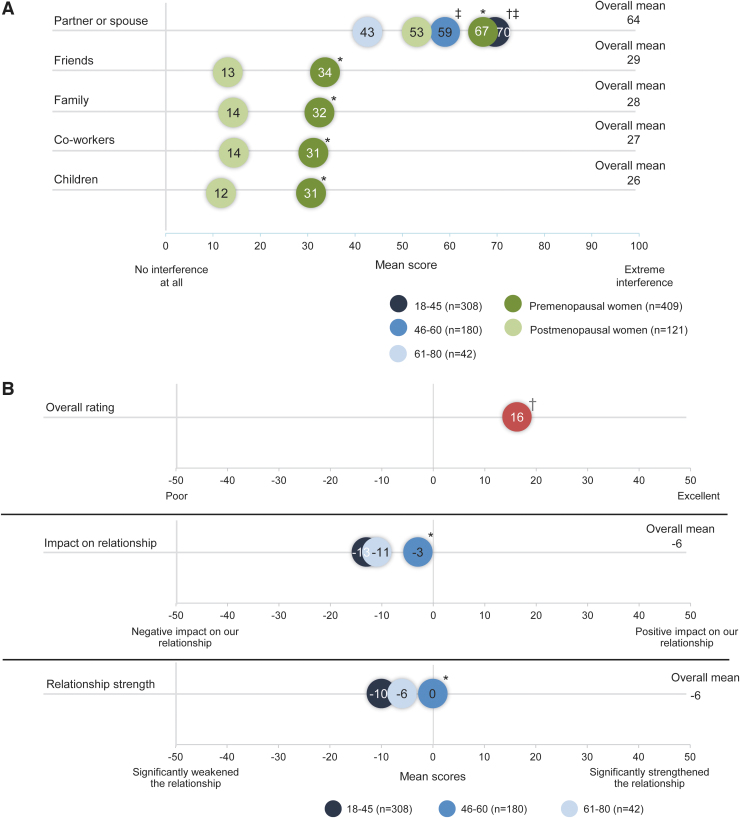

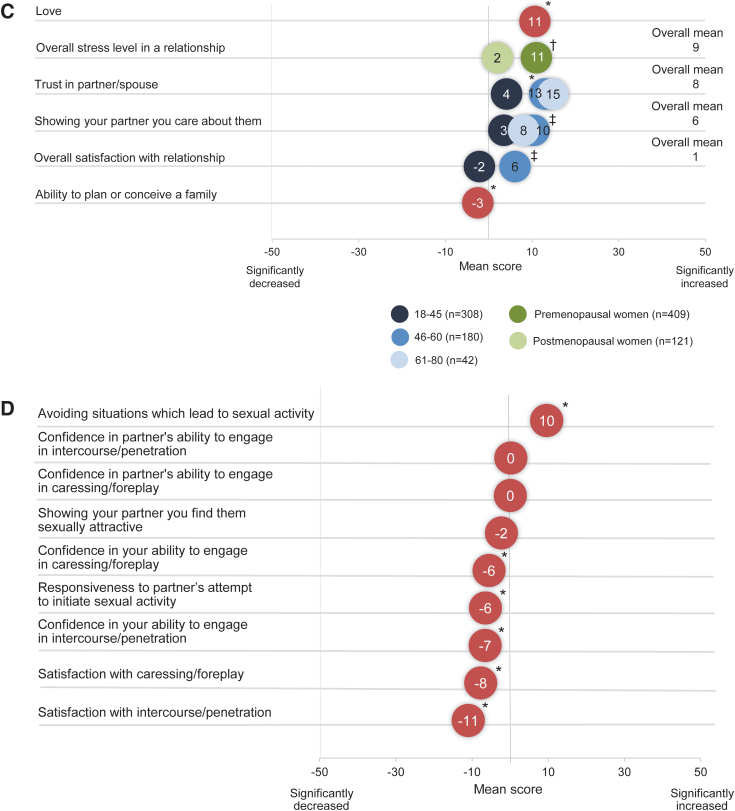
Effect of HSDD on social relationships (*n* = 530). Comparisons between the groups were analyzed using Student's *t*-tests with Bonferroni corrections as appropriate. **(A)** Impact of HSDD on social relationships by age group and menopausal status. *Statistically significantly greater than postmenopausal women. ^†^Statistically significantly greater than the 46–60 age group. ^‡^Statistically significantly greater than the 61–80 age group. **(B)** Overall impact of HSDD on partner relationship and relationship strength by age group. *Statistically significantly greater than the 18–45 age group. ^†^Statistically significantly different from zero.

Regardless of menopausal status, women with HSDD reported that relationships with their partners/spouses were impacted most (Fig. 2A). Despite the high interference in partner/spouse relationships in terms of negative impact and decreased relationship strength, participants still viewed their relationships as positive (Fig. 2B). Sexual elements in the relationship were negatively affected by HSDD and varied with menopausal status. The increase in overall stress in their relationship was significantly higher among premenopausal women compared with postmenopausal women (*p* < 0.001), and the increase in trust and overall satisfaction was also lower among premenopausal women (Fig. 2C). Consistently, participants also reported increased avoidance of situations that may lead to sexual activity (Fig. 2D).

### Impact of HSDD on mental wellness and daily activities

**FIG. 3.**
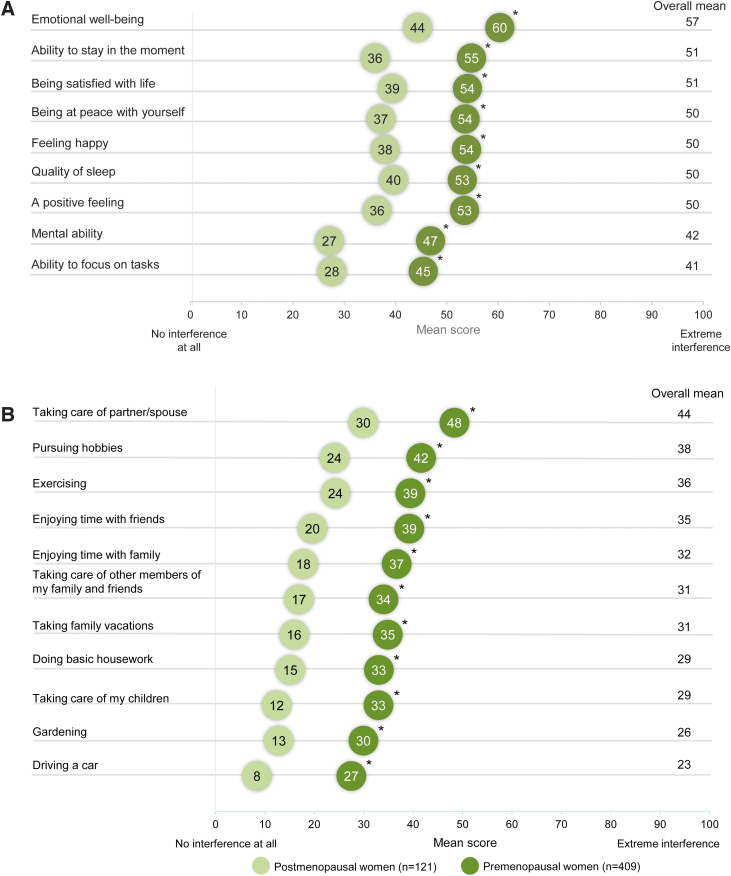
Impact of HSDD on **(A)** mental wellness by menopausal status and **(B)** daily activities by menopausal status. *Statistically significantly greater than the postmenopausal group. Comparisons between premenopausal and postmenopausal women were analyzed using Student's *t*-tests.

The effect of HSDD on overall mental well-being was assessed by measuring the degree of interference in emotional well-being, the ability to “stay in the moment,” satisfaction with life, being at peace with oneself, feeling happy, quality of sleep, positive feeling, mental ability, and ability to focus on tasks. The reliability of these endpoints was determined during the pretest evaluation, which was also developed based on input from sexual medicine experts. There was a significantly greater interference across all attributes of overall mental well-being and daily activities in premenopausal women versus postmenopausal women (*p* ≤ 0.0001) (Fig. 3A). The impact of HSDD on daily activities was also evaluated using the validated endpoints to better understand the burden of HSDD on women's lives.

Daily activities included taking care of the partner/spouse, pursuing hobbies, exercising, and enjoying time with friends, family, and others. Although the interference across daily activities was low to moderate, the impact was greater on premenopausal women compared with postmenopausal women (Fig. 3B). HSDD symptoms did not result in significant interference with work or employment (Supplementary Table S3). Regardless of age, participants with HSDD reported that within the past 6 months, ∼11.4 work hours were missed because of HSDD symptoms, suggesting minimal economic impact on women's work lives. Women aged 46–60 years were absent from work ∼19.7 hours within the past 6 months.

### Health-related quality of life

The HRQoL SF-12 score includes an MCS and physical component score (PCS).^17^ A score of 50 for both the MCS and PCS scales represents the general population norm; a score lower than 50 indicates poorer mental or physical health than the norm. Overall, participants reported an MCS of 40.6, whereas the PCS of participants (50.2) was similar to that of the general population norm. Additionally, premenopausal women reported a lower MCS (38.8) compared with postmenopausal women (46.8), whereas the PCS reported by premenopausal and postmenopausal women (50.4 vs. 49.6) were almost identical. Consistent with those results, 64.9% of premenopausal women and 40.7% of postmenopausal women had a MCS lower than that of the general population.

Among all participants, 73.2% had a normal PCS, which was similar between premenopausal (71.8%) and postmenopausal (78.0%) women. Classification of responses based on age showed that among participants with HSDD, younger women were more likely to report a lower SF-12 MCS than the general population, implying a greater impact on mental health among younger versus older women.

### Bivariate impact of HSDD on HRQoL

Considering the psychosocial factors that contribute to HSDD burden such as social relationships, mental or emotional well-being, ability to perform household or personal activities, and ability to perform at work, participants were asked to report the impact of HSDD on their overall lives. Their responses were scored on a scale of 0–100, with 0 indicating no interference in any of the factors and 100 indicating maximum interference.

Premenopausal women had significantly higher interference scores compared with postmenopausal women (63.3 vs. 45.0, *p* < 0.001) (Supplementary Fig. S4). Of note, women aged 18–45 years scored significantly higher (*p* < 0.001) than women aged 46–60 years and 61–80 years. Additionally, participants with lower scores on the SF-12 MCS and PCS reported experiencing a greater impact of HSDD symptoms (*p* < 0.001) compared with those with higher scores (data not shown).

### Multivariable regression analyses

To understand which aspects contribute toward the overall burden of HSDD and lower SF-12 MCS, two distinct multivariable linear regression models were run and analyzed. The relationship with a partner or spouse (standardized regression coefficient [β] = 0.39), mental and emotional well-being (β = 0.27), and household and personal activities (β = 0.17) were the largest contributors toward overall burden of HSDD (adjusted *R*^2^ = 0.56) (Supplementary Table S3). Consistent with the factors influencing the overall burden of HSDD, interference with the relationship with a partner (β = −0.18), mental and emotional well-being (β = −0.23), and household and personal activities (β = −0.23) had a negative impact on the SF-12 MCS (adjusted *R*^2^ = 0.28).

## Discussion

HSDD is associated with lower HRQoL, including less happiness and satisfaction with partners, and more frequent negative emotional states.^1^ As mentioned, HSDD is underdetected and undertreated. Fewer than half of patients with sexual problems seek help from physicians or initiate discussions with them. This is due to fear of self-embarrassment or concern about embarrassment to physicians. Mainly, women prefer that physicians open any such discussions with them.^1^ When asked about experience with HCPs, subjects in another recent survey reported that their PCPs inquired infrequently about their sexual health. The subjects believed that OB/GYNs asked about sexual health more frequently than PCPs.

This is possibly due to the depth and specialization of expertise and training in female reproductive health through the life course that OB/GYNs have, allowing them to be better positioned to address sexuality issues with female patients.^18^ The DSDS, the tool used in the current survey for patient inclusion, detects and diagnoses HSDD and is validated for use in general practice; it was specifically designed for HCPs who do not specialize in FSD.^19^

Treatment for HSDD often starts with programs geared to biopsychosocial elements unique to a patient's medical history and current symptoms.^20^ Cognitive behavioral therapy, mindfulness meditation training, and couples therapies have been suggested to be effective, although randomized controlled trials in women with HSDD should be performed.^21–25^ Pharmacotherapies that have been tried or utilized for HSDD include off-label bupropion and buspirone, although sufficient data are lacking for their efficacy in patients with HSDD.^2,26–29^ Off-label testosterone has shown efficacy in postmenopausal women with HSDD. However, potential serious adverse events have precluded its use in premenopausal women.^30,31^

In 2015, flibanserin (Addyi^®^; Sprout Pharmaceuticals), indicated for acquired generalized HSDD in premenopausal women, was approved by the FDA. Flibanserin (a postsynaptic 5-hydroxytryptamine 1A agonist and 2A antagonist) decreases serotonin levels and increases dopamine and norepinephrine levels, neurotransmitters that affect sexual desire.^15,32^ It is believed that flibanserin affects brain function through enhancement of excitatory elements and decreasing inhibitory responses to sexual cues.^29^

Bremelanotide (Vyleesi^®^; AMAG Pharmaceuticals), a novel cyclic 7-amino acid melanocortin-receptor agonist with high affinity for the melanocortin-4-receptor, was approved by the FDA in June 2019 and is also indicated for the treatment of premenopausal women with acquired generalized HSDD.^15,33^ Results from preclinical studies indicated that bremelanotide acts on the physiological and neurobiological components of female sexual function, and it has the potential to modulate neural pathways involved in sexual desire and arousal in women with HSDD.^34^

The efficacy and safety of bremelanotide, taken on demand over 24 weeks, demonstrated statistically significant and clinically meaningful improvements in low sexual desire and related distress in two identically designed, phase 3, randomized, double-blind, placebo-controlled studies with a 6-month open-label extension.^35,36^

### Principal findings

In the current survey, HSDD had a significant negative impact on sexual and mental health, social relationships, and general well-being. The overall burden associated with HSDD was greater in premenopausal women than in postmenopausal women despite the generally higher prevalence of HSDD in postmenopausal women.^5,37^ While we cannot conclude that HSDD was the sole contributor to the negative impact on sexual and mental health in the reported bivariate results, HSDD likely played a role as all respondents qualified as having the condition based on either the DSDS or an HSDD diagnosis.

As measured by the SF-12 MCS, premenopausal women experienced a greater impact of HSDD on their mental health than did postmenopausal women. HSDD more profoundly affected partner/spouse relationships and the mental/emotional well-being of premenopausal women than postmenopausal women. Premenopausal women also experienced greater interference in their daily activities. In contrast, low sexual function scores had no significant impact on physical health regardless of menopausal status and/or age. The frequency of sexual activities did not differ significantly between premenopausal and postmenopausal women with HSDD. This result is consistent with findings that women may engage in sexual relations with their partners despite the absence of sexual desire or interest.^38^

Multivariable regression analyses showed that the overall burden of HSDD was driven by interference with the relationship with the participant's partner, mental and emotional well-being, and household and personal activities and that the burden of HSDD was associated with lower SF-12 MCS (relative to the norms) regardless of menopausal status.

Consistent with other studies, a substantial proportion of participants had not spoken or delayed speaking to an HCP about their HSDD symptoms; the majority of interactions on the subject were initiated by participants.^1,7,8^

### Clinical implications

Many participants reported that their clinicians were unaware of HSDD, suggesting that there is insufficient education on HSDD among physicians who manage patients with this disorder. The majority of participants were prescribed treatments with no evidence of clinical efficacy.

The results of this study demonstrate that despite its significant burdens for both premenopausal and postmenopausal women, HSDD is underrecognized and undertreated, underscoring substantial associated unmet health care needs. Because HSDD may manifest in several ways, such as feelings of low self-esteem, grief, incompetence, or loss, it is important that HCPs rule out these symptoms as the primary cause of low sexual desire.^2,3,39^ Validated screening tools, such as the DSDS, and educational efforts can help HCPs initiate sexual health conversations with their patients, decrease the time needed for a detailed patient history, make an accurate diagnosis, and begin treatments that have known and proven efficacy.^13^

### Research implications

This study is the first to quantitate the differences between premenopausal and postmenopausal women in the HRQoL burden of HSDD and should therefore be replicated. The results also indicate the need for additional research on the causes and correlates of HSDD among both premenopausal and postmenopausal women, and the types of educational approaches that could foster awareness and discussion with their HCPs. It is also important to develop and test educational materials for HCPs to assist them in recognizing the extent and burden of HSDD to improve diagnosis and management.

### Strengths and limitations

Web-based surveys may have certain advantages such as speed and cost of data collection as well as data quality. However, we understand that they may be biased by low and selective participation^40^; while this aspect may be a limitation in some surveys, it may be less so in the current survey.

Additionally, there is limited guidance with little consensus regarding the optimal reporting of survey research. Although some key criteria are usually reported by authors publishing this research in peer-reviewed journals, many key criteria are underreported. As in other areas of research, poor reporting compromises both transparency and reproducibility. Therefore, there is a need for a well-developed reporting guideline for survey research. Possibly an extension of the guideline for observational studies in epidemiology (The Strengthening the Reporting of Observational Studies in Epidemiology) would provide a reasonable structure, ensuring complete reporting with an improved review and interpretation of the results.^41^

Nevertheless, the methodology of cross-sectional survey-based studies has been used to document the burden of illness in patients across diverse therapeutic areas, including celiac disease, depression, and peripheral/central neuropathic pain.^42–44^ These and other studies provide evidence that patient-reported surveys are effective in determining the burden of illness for medical conditions.

In this study, we used not only a customized web-based survey but also the validated FSFI and SF-12 questionnaires to evaluate the burden of HSDD. However, other potential limitations of the study are that participants were primarily white and were required to be in a stable, monogamous relationship of at least 6 months of duration. Thus, the results of the current survey are not generalizable, and additional research needs to be conducted to discover and evaluate the findings across other types of relationships and more diverse populations.

## Conclusions

This comprehensive cross-sectional study assessed the burden of illness associated with HSDD in women, demonstrating its pervasive negative effects on several aspects of women's lives through multiple assessment instruments. The impact was consistently and significantly greater in premenopausal women than in postmenopausal women.

## Supplementary Material

Supplemental data

Supplemental data

Supplemental data

Supplemental data

Supplemental data

Supplemental data

Supplemental data
