# Supplementary material for: Assessing the Burden of Illness Associated with Acquired Generalized Hypoactive Sexual Desire Disorder
Source: J Womens Health (Larchmt). 2022 May 16;31(5):715–25. doi: 10.1089/jwh.2021.0255 (PMC9133974; doi:10.1089/jwh.2021.0255)
Supplement: Supplemental data [file Suppl_TableS1.docx]

**SUPPLEMENTARY TABLE 1. Decreased Sexual Desire Screener**

| 1. In the past, was your level of sexual desire or interest good and satisfying to you? |
| --- |
| 1. Has there been a decrease in your level of sexual desire or interest? |
| 1. Are you bothered by your decreased level of sexual desire or interest? |
| 1. Would you like your level of sexual desire or interest to increase? |
| 1. Please check all the factors that you feel may be contributing to your current decrease in sexual desire or interest:    1. An operation, depression, injuries, or other medical condition    2. Medication, drugs, or alcohol you are currently taking    3. Pregnancy, recent childbirth, menopausal symptoms    4. Other sexual issues you may be having (pain, decreased arousal or orgasm)    5. Your partner’s sexual problems    6. Dissatisfaction with your relationship or partner    7. Stress or fatigue |

From Clayton AH, Goldfischer ER, Goldstein I, DeRogatis L, Lewis-D’Agostino DJ, Pyke R. Validation of the Decreased Sexual Desire Screener (DSDS): a brief diagnostic instrument for generalized acquired female hypoactive sexual desire disorder. *J Sex Med*. 2009;6(3):730-738. © 2009 International Society for Sexual Medicine, with permission from Elsevier.

The Decreased Sexual Desire Screener (DSDS) is a 5-item self-report questionnaire that has been validated as a screening tool to assist internists, family physicians, and gynecologists without expertise in sexual medicine to make an accurate diagnosis of acquired, generalized hypoactive sexual desire disorder (HSDD).^1^ The first 4 questions establish the presence of decreased sexual desire and indicate that further assessment is required.^1^ A “yes” response to the first 4 questions indicates HSDD; a “yes” response to any part of question 5 indicates low sexual desire caused by other reasons, including comorbid conditions. Participants who answered a “yes” response to any part of question 5 of the DSDS were excluded, and the exclusion included women who had undergone a bilateral oophorectomy or hysterectomy, or who had decreased sexual desire due to an operation, depression, injuries, or other medical condition; medications, drugs, or alcohol; pregnancy, recent childbirth, or menopausal symptoms; other sexual issues, such as pain, decreased arousal or orgasm; partner’s sexual problems; dissatisfaction with their relationship or partner; and/or stress or fatigue.
