# Supplementary material for: Assessing the Burden of Illness Associated with Acquired Generalized Hypoactive Sexual Desire Disorder
Source: J Womens Health (Larchmt). 2022 May 16;31(5):715–25. doi: 10.1089/jwh.2021.0255 (PMC9133974; doi:10.1089/jwh.2021.0255)
Supplement: Supplemental data [file Suppl_FigS1.docx]

**SUPPLEMENTARY FIGURE 1.** Patient-reported reasons for the delay in their diagnosis of HSDD. HSDD, hypoactive sexual desire disorder.
