# Supplementary material for: Assessing the Burden of Illness Associated with Acquired Generalized Hypoactive Sexual Desire Disorder
Source: J Womens Health (Larchmt). 2022 May 16;31(5):715–25. doi: 10.1089/jwh.2021.0255 (PMC9133974; doi:10.1089/jwh.2021.0255)
Supplement: Supplemental data [file Suppl_TableS2.docx]

**SUPPLEMENTARY TABLE 2. Initiation of conversations about HSDD symptoms**

|  | **Doctor/Therapist Who Was First Approached,**  **n (% participants)**  **(N=324)** | **Doctor/Therapist Type Who First Diagnosed Patient With Decreased Sexual Desire,**  **n (% participants)**  **(N=324)** |
| --- | --- | --- |
| Gynecologist | 164 (51) | 158 (49) |
| PCP | 146 (45) | 110 (34)^a^ |
| Sex therapist | 23 (7) | 30 (9) |
| Therapist who is not a sex therapist | 24 (7) | 10 (3) |
| Psychologist | 18 (5) | 19 (6) |
| Psychiatrist | 14 (4) | 12 (4) |
| Other | 3 (1) | 2 (1) |

HSDD, hypoactive sexual desire disorder; PCP, primary care provider.

^a^21% of the women who initially approached a PCP were ultimately diagnosed by a gynecologist.
