# Supplementary material for: Assessing the Burden of Illness Associated with Acquired Generalized Hypoactive Sexual Desire Disorder
Source: J Womens Health (Larchmt). 2022 May 16;31(5):715–25. doi: 10.1089/jwh.2021.0255 (PMC9133974; doi:10.1089/jwh.2021.0255)
Supplement: Supplemental data [file Suppl_FigS3.docx]

**SUPPLEMENTARY FIGURE 3.** Treatment options recommended by health care provider (n=340).
