# Supplementary material for: Assessing the Burden of Illness Associated with Acquired Generalized Hypoactive Sexual Desire Disorder
Source: J Womens Health (Larchmt). 2022 May 16;31(5):715–25. doi: 10.1089/jwh.2021.0255 (PMC9133974; doi:10.1089/jwh.2021.0255)
Supplement: Supplemental data [file Suppl_TableS3.docx]

**SUPPLEMENTARY TABLE 3. Factors contributing to overall burden of HSDD and SF-12 mental component score**

| **Dependent Variable: Overall Burden of HSDD^a^** | **Standardized Coefficient** | ***P*-value** | **95% Confidence Interval** | |
| --- | --- | --- | --- | --- |
|  |  |  | **Lower Bound** | **Upper Bound** |
| Relationship with partner or spouse | 0.39 | <.005 | 0.28 | 0.45 |
| Mental and emotional well-being | 0.27 | <.005 | 0.15 | 0.38 |
| Household and personal activities | 0.17 | .02 | 0.02 | 0.32 |
| Work and/or school | 0.07 | .30 | -0.05 | 0.16 |
| Relationship with family members | 0.06 | .52 | -0.11 | 0.22 |
| Relationship with friends | 0.02 | .86 | -0.14 | 0.16 |
| Relationship with co-workers | -0.06 | .49 | -0.20 | 0.10 |
| Relationship with children | -0.07 | .39 | -0.20 | 0.08 |
| **Dependent Variable: SF-12 MCS^b^** | **Standardized Coefficient** | ***P-*value** | **95% Confidence Interval** | |
|  |  |  | **Lower Bound** | **Upper Bound** |
| Relationship with your partner or spouse | -0.18 | <.005 | -0.11 | -0.02 |
| Mental and emotional well-being | -0.23 | <.005 | -0.15 | -0.03 |
| Household and personal activities | -0.23 | .02 | -0.17 | -0.02 |
| Work and/or school | -0.09 | .28 | -0.08 | 0.02 |
| Relationship with family members | 0.29 | .02 | 0.02 | 0.19 |
| Relationship with friends | -0.03 | .78 | -0.09 | 0.07 |
| Relationship with co-workers | 0.07 | .50 | -0.05 | 0.10 |
| Relationship with children | -0.04 | .70 | -0.09 | 0.06 |

HSDD, hypoactive sexual desire disorder; MCS*,* mental component score; SF-12, 12-Item Short Form Survey.

^a^Adjusted *R^2^*=0.56.

^b^Adjusted *R^2^*=0.28.

For both multivariable linear regression models, mean scores for relationship with partner/spouse, mental and emotional well-being, household and personal activities, work and/or school, relationship with family members, friends, children, and co-workers were calculated and included as independent variables in the analyses. The following control variables were included in the regression analyses: age, race, education, marital status, and social desirability bias that were measured by egoistic response tendencies and moralistic response tendencies.
